# Disrupting the Algorithm: The Role of Medical Professionals in TikTok’s Post-traumatic Stress Disorder Conversations

**DOI:** 10.7759/cureus.94991

**Published:** 2025-10-20

**Authors:** Lindsey Nichols, Jennifer Frazee, Brittany Quinn, Mark Payton, Rachel Linger

**Affiliations:** 1 Medicine, Rocky Vista University College of Osteopathic Medicine, Englewood, USA; 2 Biomedical Sciences, Rocky Vista University College of Osteopathic Medicine, Englewood, USA

**Keywords:** engagement, medical professional, ptsd, social media, tiktok

## Abstract

Introduction: Social media platforms have become important spaces for sharing information and personal experiences related to mental health conditions, including post-traumatic stress disorder (PTSD). TikTok, in particular, has rapidly gained popularity among users aged 13 to 24 years old, who increasingly seek mental health content. This study explores engagement patterns with PTSD-related TikTok videos, comparing audience interactions with content created by medical professionals and non-medical creators.

Methods: A total of 200 English-language TikTok videos were analyzed. Creators were classified as medical professionals or non-medical professionals based on profile information, LinkedIn verification, and institutional affiliations. Engagement metrics, including views, likes, comments, and shares, were collected and analyzed using t-tests to assess differences between creator types.

Results: Non-medical creators produced the majority of content (n= 168; 84%), while medical professionals contributed 16% (n= 32). Cumulative engagement metrics, total views, comments, likes, and shares, did not differ significantly between groups. However, videos by non-medical creators received significantly more likes per day (p=0.04), reflecting faster initial engagement. In contrast, videos by medical professionals accumulated similar overall engagement over a longer period, suggesting steady and sustained interaction.

Discussion: These findings challenge the assumption that medical professionals are at a disadvantage on platforms like TikTok. Despite non-medical creators generating more immediate engagement, medical professionals can reach comparable audiences over time, offering an opportunity to disseminate accurate, evidence-based information. The results emphasize the importance of medical professionals adopting platform-specific strategies to increase their visibility and credibility in digital spaces. This study advances understanding of PTSD discourse on social media and highlights the critical role digital platforms play in shaping public perceptions of mental health.

## Introduction

Social media has become a dominant source of health information, particularly for younger populations seeking accessible, peer-driven content. Platforms such as TikTok, known for its short-form videos, algorithm-driven content curation, and high interactivity, have emerged as central spaces for health discourse, including mental health [1]. TikTok is especially popular among youth and young adults. In 2023, nearly 63% of U.S. teenagers reported using the platform, with the highest activity among individuals aged 13-24 years old [2]. While TikTok’s minimum age requirement is 13, studies suggest that even younger users access the platform, often circumventing formal restrictions [3].

This rise in digital health-seeking behaviors presents both opportunities and challenges. On the one hand, TikTok can increase awareness, reduce stigma, and foster peer support around mental health [4,5]. On the other hand, concerns about misinformation, oversimplification, and emotionally charged narratives are well-documented [6,7]. Emotional storytelling, while engaging, can sometimes reinforce maladaptive coping or normalize symptoms in ways that discourage treatment. For example, social media messages highlighting distress or suffering often spread rapidly, amplifying emotional reactions without offering constructive coping strategies [8].

Post-traumatic stress disorder (PTSD) is a complex psychiatric condition characterized by intrusive thoughts, avoidance, negative mood, and hyper-arousal following exposure to trauma [9]. Despite its clinical seriousness, PTSD-related content is highly visible on TikTok under hashtags such as #PTSD, #trauma, and #CPTSD. CPTSD, or complex post-traumatic stress disorder, is a condition that encompasses core symptoms of PTSD but also includes additional features such as difficulty regulating emotions, persistent feelings of worthlessness or shame, and challenges in maintaining relationships or feeling connected to others [10]. The majority of trauma-related TikTok videos are created by non-clinicians, with content largely emphasizing personal experiences and emotional appeal rather than evidence-based education, and licensed professionals remain underrepresented [6,7].

A critical aspect of understanding health communication on TikTok involves examining platform-specific affordances, the features that shape how information is created, shared, and received [11]. Previous studies have shown that TikTok's algorithm favors engagement-rich, emotionally resonant content over traditional markers of expertise [12-14], which poses unique challenges for medical professionals, whose content may be less visually dynamic or emotionally provocative. Without adapting their strategies to the platform’s norms (e.g., storytelling, music/audio use), medical creators may be less visible, even when their content is accurate [15].

Engagement metrics on TikTok include both passive and active forms of interaction. Views represent passive exposure, while likes, comments, and shares indicate active engagement and user response. These metrics are not interchangeable; a video may have many views but low engagement, suggesting limited impact or resonance [16]. Understanding how these metrics differ between medical and non-medical creators can provide insights into both reach and influence.

Given these dynamics, this study asks how PTSD is represented on TikTok and how medical professionals are situated within that discourse. Specifically, we examine what proportion of PTSD-related TikTok videos are created by medical versus non-medical professionals and how engagement metrics such as views, likes, comments, and shares differ across these groups. By addressing these research questions, we aim to explore differences, acknowledging that content dynamics on TikTok are shaped by both creator identity and platform-specific norms. This study contributes to a growing body of work on digital health communication, emphasizing the need for medical professionals to adopt platform-savvy strategies without compromising accuracy or ethics. This study aims to examine the proportion of PTSD-related TikTok videos created by medical versus non-medical professionals and compare engagement metrics, including views, likes, comments, and shares, between these groups.

## Materials and methods

In a cross-sectional observational study, a sample of 206 TikTok videos was collected on November 16, 2024, using the search term “PTSD.” The videos in this convenience sample were the first 206 retrieved by the TikTok algorithm, using a newly created account to avoid algorithm bias. Videos were excluded if they were in languages other than English, unrelated to PTSD, or duplicates. Based on these criteria, six videos were removed, resulting in a final sample of 200 videos for analysis. 

Engagement metrics for each video, including views, likes, comments, shares, and the video’s creation date, were recorded. The engagement metrics were used to capture both passive and active forms of audience interaction. Views represent the total number of times a video was displayed on a user’s screen, reflecting overall exposure or reach. Likes indicate a user’s positive reaction to the video, serving as a quick, low-effort form of endorsement. Comments represent written responses posted by users and are considered a more active form of engagement, reflecting direct audience interaction and discussion. Shares refer to the number of times users distributed the video to others via TikTok's sharing function, indicating that the content resonated enough to be disseminated within or beyond the platform. The video creation date was used to calculate how long each video had been online at the time of data collection, allowing for the determination of daily engagement rates.

To calculate average daily engagement, total engagement was documented for the data collection date and divided by the number of days the video had been online. Additionally, the user profiles of each video were analyzed to classify each creator as a “medical professional” or “non-medical professional.” The "medical professional" category was further subdivided by specific titles, including but not limited to DO/MD, nurse practitioners, nurses, psychologists, social workers, and therapists. Each medical professional was verified on LinkedIn or through institutional affiliations when possible. Unverified medical professionals were recorded under their claimed profession, with their verification status noted. An additional “other medical professional” category captured creators who have advanced scientific or medicine-adjacent expertise such as those with PhDs in pharmacology or pharmaceutical sciences, neuroscience, pharmacists or physician assistants. The decision to count these creators as medical professionals rather than non-medical professionals was made due to their high level of education in a field related to mental health. 

Ethical statement

This project was reviewed by the Rocky Vista University Institutional Review Board (IRB) on October 31st, 2024 and determined to be Not Human Subjects Research under 45 CFR 46.102(e). The study analyzed publicly available TikTok posts without interacting with users, recording direct identifiers, or attempting to determine the age of creators; therefore, IRB approval and informed consent were not required. Only publicly accessible content was analyzed, and no usernames, handles, profile photos, or other information that could reasonably identify individuals, particularly minors, will be published. Engagement data (views, likes, comments, shares) are reported only in aggregate. All data collection complied with TikTok’s Terms of Service, and no prohibited scraping or automated data extraction methods were used. Raw data files are stored on secure, access-controlled drives and will be retained for a period consistent with institutional policy. Medical professional status was verified using publicly available information, including LinkedIn profiles and institutional affiliations. 

Statistical methods

All statistical analyses were performed using SAS Version 9.4 (SAS Institute, Cary, NC). Since the engagement metrics (total comments/likes/shares and comments/likes/shares per day) for produced videos are meaningfully numeric, comparisons of the means of medical professionals to non-medical professionals were conducted with independent t-tests. Since the sample sizes are large, normality was assumed using the Central Limit Theorem. Homogeneity of variances was examined using folded F-tests. If the variances appeared to be statistically unequal, a Satterthwaite degree-of-freedom approximation was used to mitigate this issue. Due to multiple tests being conducted, p-values were adjusted using the Holm-Bonferroni method. Statistical significance was determined as p < 0.05. 

## Results

This study examined the distribution of TikTok videos about PTSD by creator type. To determine creator identity, user profiles were reviewed, and claims of medical professional status were verified via LinkedIn and institutional affiliations. Medical professionals produced 16% (n= 32) of PTSD-related posts on TikTok, while non-medical professionals accounted for 84% (n=168) (Table 1). The credentials of 87.5% (28/32) of medical professionals were successfully verified (Table 2). 

**Table 1:** Distribution of content creator identities

Credential	Frequency	Percent
Medical Professional	32	16%
DO/MD	5	2.50%
Nurse Practitioner	3	1.50%
Nurse	1	0.50%
Psychologist	5	2.50%
Social Worker	2	1.00%
Therapist	8	4.00%
Other	8	4.50%
Non-medical Professional	168	84%

**Table 2:** Verification status of credentials

Verification Status	Number of Creators (n= 32)	Percentage (%)
Verified	28	87.5
Not Verified	4	12.5

**Figure 1:**
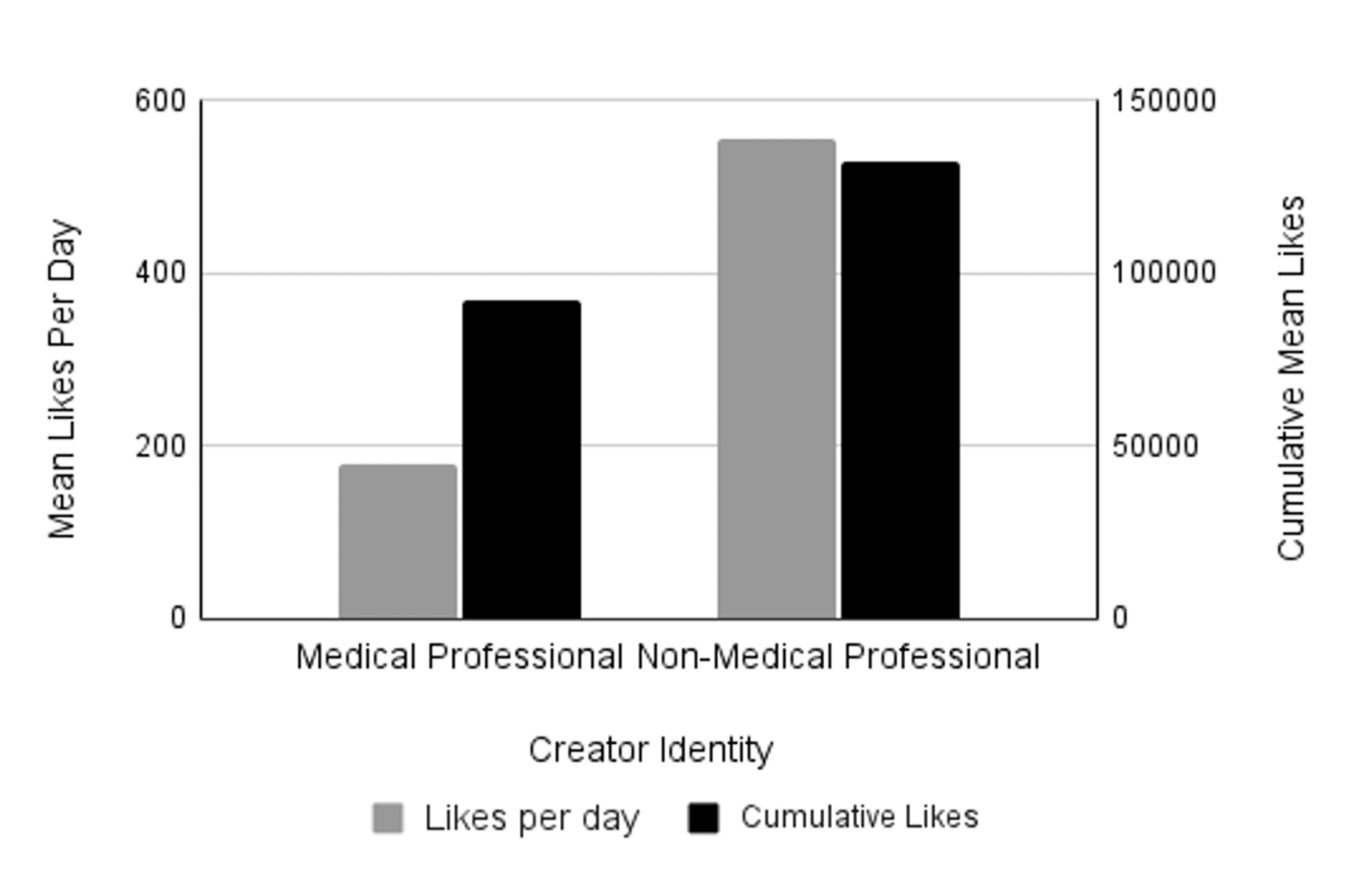
Mean number of likes by creator identity The left vertical axis measures mean likes per day (gray bars) and the right vertical axis measures mean cumulative likes (black bars). Videos created by non-medical professionals received significantly more likes per day than videos created by medical professionals (p=0.04; p-value adjusted using the Holm-Bonferroni method). There was no significant difference in the cumulative mean number of likes between videos created by medical professionals or non-medical professionals (p=0.39).

**Figure 2:**
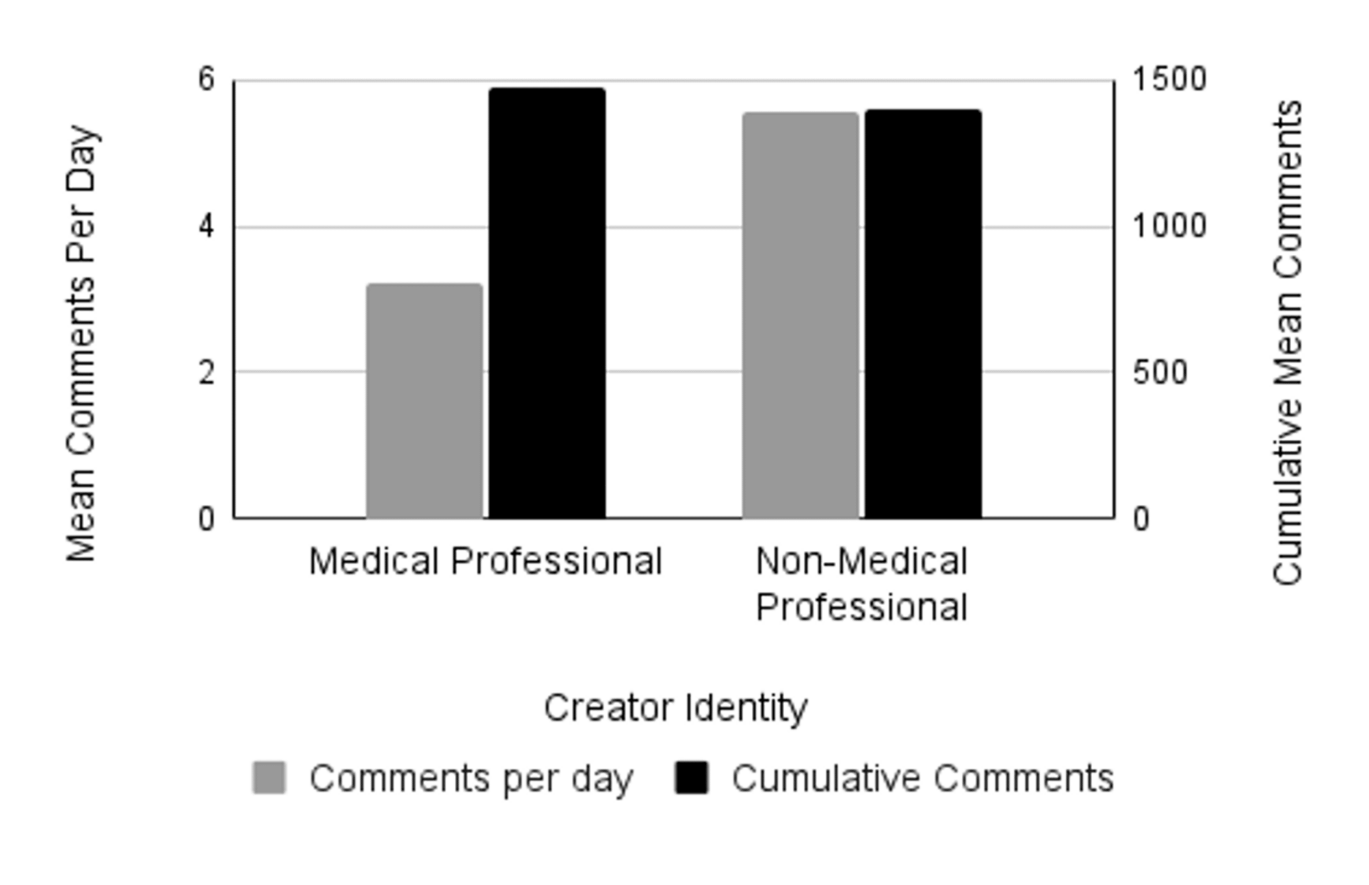
Mean number of comments by creator identity The left vertical axis measures mean comments per day (gray bars) and the right vertical axis measures mean cumulative comments (black bars). There was no significant difference in the comments per day (p=0.26) or cumulative mean number of comments (p=0.91) between videos created by medical professionals or non-medical professionals.

**Figure 3:**
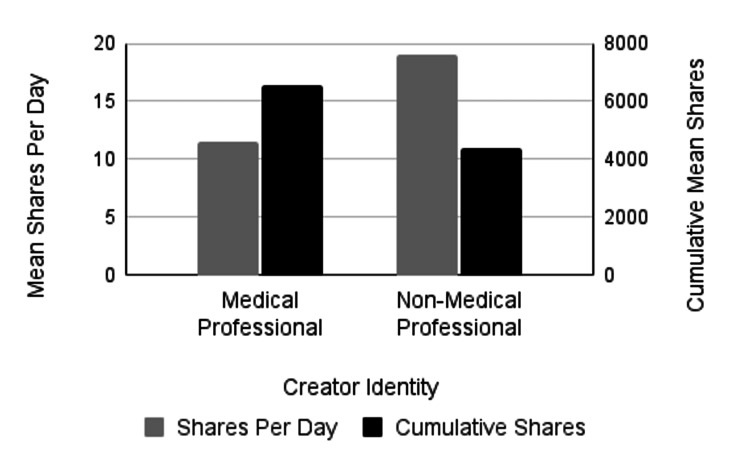
Mean number of shares by creator identity The left vertical axis measures mean shares per day (gray bars) and the right vertical axis measures cumulative mean shares (black bars). There was no significant difference in the mean number of shares per day (p=0.25) or mean cumulative shares (p=0.53) between videos created by medical professionals or non-medical professionals.

**Figure 4:**
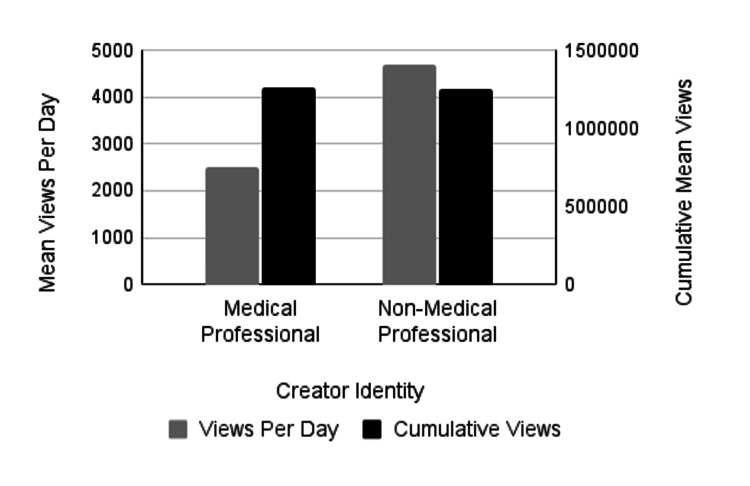
Mean number of views by creator identity The left vertical axis measures mean views per day (gray bars) and the right vertical axis measures mean cumulative views (black bars). There was no significant difference in the mean number of views per day (p=0.15) or mean cumulative views (p=0.99) between videos created by medical professionals or non-medical professionals.

The analysis also assessed whether PTSD-related videos created by non-medical professionals garnered higher engagement, measured by views, likes, comments, and shares, compared to those produced by medical professionals; however, the results did not consistently support this. To determine differences in engagement between content created by medical professionals and non-medical professionals, we compared the cumulative number of likes, comments, shares, and views. TikTok videos created by medical professionals averaged 92,372.6 cumulative likes, while TikTok videos created by non-medical professionals averaged 132,404 cumulative likes. This trend in more likes for non-medical professionals' content was not statistically significant (p=0.39) (Figure 1; Table 3). TikTok videos created by medical professionals averaged 1,471.5 cumulative comments, while TikTok videos created by non-medical professionals were very similar, with an average of 1,394.9 cumulative comments, which was not statistically significant (p=0.91) (Figure 2; Table 3). Medical professionals' videos had an average of 6,554.3 shares compared to 4,397.9 shares for non-medical professionals, though this difference was not statistically significant (p=0.53) (Figure 3; Table 3). It is noteworthy that this is the only engagement metric in which medical professionals demonstrated considerably higher levels of engagement compared to non-medical professionals. Lastly, the average number of views for medical professionals' videos was 1,260,482, while non-medical professionals' videos had 1,253,033 views, with no statistically significant difference (p=0.99) (Figure 4; Table 3). While we found no significant difference in these cumulative metrics, it is important to note that our sample size of medical professionals (n=32) was much smaller than the sample of non-medical professionals (n=168). Additionally, 12.5% of medical professionals’ identities could not be identified, which may have influenced the findings. 

**Table 3:** Comparison of mean engagement metrics between medical and non-medical professionals' TikTok videos

Engagement Metric	Medical Professionals	Non-medical Professionals	Standardized Effect Size	Effect Confidence Interval	p-value
	(n=32)	(n=168)			
Average Likes	92,372.6	132,404	-0.16	(-0.54, 0.22)	0.39
Average Comments	1,471.5	1,394.9	0.02	(-0.36, 0.40)	0.91
Average Shares	6,554.3	4,397.9	0.17	(-0.37, 0.71)	0.53
Average Views	1,260,482	1,253,033	0.00	(-0.52, 0.51)	0.99

To assess differences in engagement between content created by medical and non-medical professionals, likes, comments, shares, and views per day were compared. There was no significant difference in the average number of days the TikTok videos had been online at the time of data collection, with videos by medical professionals averaging 477.5 days and those by non-medical professionals averaging 483.5 days (p = 0.9411). The analysis examined whether videos created by non-medical professionals received higher engagement per day, which was partially supported by the data. TikTok videos created by medical professionals averaged 3.22 comments and 2,514.3 views per day, whereas videos by non-medical professionals averaged 5.56 comments and 4,683 views per day (Figures 2, 4, respectively; Table 4). Despite averaging nearly twice the number of views and comments per day, these differences were not statistically significant (p = 0.26; p = 0.15). Videos created by non-medical professionals averaged 19.01 shares per day, compared to 11.5 shares per day for medical professionals. Although non-medical professionals received a higher number of shares, the difference was not statistically significant (p = 0.25) (Figure 3; Table 4). Additionally, videos by non-medical professionals received significantly more likes per day than those by medical professionals (p = 0.04; p-value adjusted using the Holm-Bonferroni method), with medical professionals averaging 178.6 likes per day and non-medical professionals averaging 556.0 likes per day (Figure 1; Table 4). Most TikTok videos about PTSD in our sample were created by non-medical professionals. Interestingly, we found no significant differences between the two groups in the majority of cumulative and daily engagement metrics. Additionally, there was no significant difference in how long the videos had been posted at the time of data collection. 

**Table 4:** Comparison of engagement per day between medical and non-medical professionals *p< 0.05 is considered statistically significant.

Engagement Metric	Medical Professionals	Non-medical Professionals	Standardized Effect Size	Effect Confidence Interval	p-value
	(n=32)	(n=168)			
Average Likes/Day	178.6	556	-0.27	(-0.47, -0.07)	0.04*
Average Comments/Day	3.22	5.56	-0.14	(-0.37, 0.11)	0.26
Average Shares/Day	11.5	19.01	-0.14	(-0.39, 0.11)	0.25
Average Views/Day	2,514.3	4,683	-0.20	(-0.48, 0.08)	0.15

## Discussion

Principle results

This study highlights a crucial dynamic in the dissemination of PTSD-related content on TikTok. Despite the dominance of non-medical professionals in creating this content (84%; n=168 of the sample), medical professionals have the potential to achieve comparable engagement levels. The analysis explored whether videos by non-medical professionals would consistently outperform those of medical professionals across all engagement metrics; however, the findings indicate a more nuanced pattern.

The most notable difference was in the rate of engagement. Non-medical professional videos received significantly more likes per day, suggesting a pattern of rapid, viral-like dissemination. However, the total cumulative likes were not significantly different between the two groups. To explore whether the time a video had been live might account for these differences, we conducted a t-test, and the results were strikingly insignificant, indicating no meaningful difference in video age between medical and non-medical creators. This reinforces the interpretation that differences in engagement rate are more likely driven by content characteristics rather than duration on the platform. 

These findings carry important implications for the role of medical professionals in digital mental health discourse. While non-medical professionals dominate in sheer volume, our data suggests that medical professionals have an opportunity to engage audiences at a comparable level if they adopt platform-specific strategies. By increasing their presence, embracing accessible communication styles, and leveraging TikTok’s algorithmic trends, medical professionals can play a critical role in steering online conversations toward accurate, evidence-based information [1]. Research suggests that incorporating trending audio and features on TikTok can enhance audience engagement by creating shared experiences and encouraging interaction, which can amplify the reach and impact of content [17]. This emphasizes the importance of adapting communication styles to platform norms while maintaining evidence-based messaging [18].

These findings carry broader implications for digital mental health communication. Despite producing fewer videos, medical professionals have a clear opportunity to influence online discourse, particularly if they tailor their communication style to meet the norms of the platform. This includes simplifying language, incorporating storytelling, and responding to TikTok trends to make evidence-based content more approachable and resonant with viewers. 

The importance of this shift is particularly pronounced in the context of adolescent mental health, a group that comprises a significant portion of TikTok’s user base. Although research shows that adolescents view healthcare professionals as trustworthy sources of information, they often encounter barriers to engagement, such as perceived inaccessibility or lack of relatability [19]. By meeting adolescents where they are on digital platforms and communicating in a format they find appealing, medical professionals can help bridge this gap. While our study did not specifically measure adolescent engagement, the high prevalence of PTSD content on TikTok and the platform’s dominant youth demographic highlight the potential public health impact. Effective outreach may not only improve knowledge and reduce stigma but also encourage help-seeking behaviors among young people [20]. 

Strengths

This study has several important strengths. It analyzes a substantial and contemporary sample of PTSD-related TikTok videos (n=200), capturing content from a platform that is rapidly shaping youth mental health discourse. By distinguishing between medical and non-medical creators and verifying credentials for the majority of medical professionals, the study adds rigor and credibility to the classification process. Incorporating multiple engagement metrics, including views, likes, comments, and shares, while also calculating average daily engagement, allows for a more nuanced understanding of audience interaction over time, rather than relying solely on cumulative totals. Focusing on PTSD, a clinically significant yet underexplored topic in digital spaces, provides insight into how sensitive mental health topics are represented and engaged with online. Additionally, using a newly created account to collect videos helps reduce algorithmic bias, increasing the generalizability of the findings across typical TikTok user experiences. 

Limitations

This research is subject to several inherent limitations, and the findings should be interpreted in light of certain methodological constraints. The lack of longitudinal data prevents us from fully characterizing engagement trends over time, and our classification of medical professionals, based on LinkedIn verification and institutional affiliations, may not capture all qualified experts. Additionally, we focused solely on engagement metrics without evaluating the accuracy of the content itself, a critical factor in assessing the impact of mental health information on TikTok. 

Future research could address these gaps by systematically evaluating the accuracy of PTSD-related TikTok videos alongside engagement metrics. Additionally, longitudinal studies could track engagement trends over time, while examining how exposure to accurate versus inaccurate content influences behaviors such as help-seeking, symptom normalization, or stigma. Qualitative methods, such as surveys or interviews, could provide further insight into how viewers interpret and respond to these videos. 

Despite these limitations, our findings highlight a clear opportunity: medical professionals are not inherently disadvantaged in social media engagement. With strategic adaptations, they can extend their reach and influence comparable to that of non-medical professionals. The next challenge is developing clear guidelines for presenting evidence-based content in digital spaces to maximize its impact, as no current framework exists to guide medical professionals in achieving these goals. 

## Conclusions

This study highlights the significant role that non-medical professionals play in shaping PTSD-related discourse on TikTok. The analysis examined whether videos by non-medical professionals would receive higher engagement across all metrics; while this was not fully supported, their content did accumulate more likes per day, indicating a pattern of rapid initial engagement. Overall engagement between medical and non-medical professionals did not differ significantly, suggesting that medical professionals can reach comparable audiences with sustained presence and strategic content creation. These findings underscore the importance of understanding the factors that drive engagement in online mental health discourse. Given the increasing reliance on social media for health information, it is essential for medical professionals to explore strategies to enhance their presence and engagement on platforms such as TikTok. Future research should investigate how content accuracy, thematic trends, and the dissemination of misinformation influence audience engagement and public perceptions of PTSD. 
